# Academic leadership in physician assistant/associate medical education: a cross-sectional analysis of the association with doctoral degree, gender, and minority status

**DOI:** 10.1186/s12909-022-03817-6

**Published:** 2022-11-23

**Authors:** Lucy W. Kibe, Gerald Kayingo, Katrina M. Schrode, Alicia Klein

**Affiliations:** 1 Charles R. Drew University of Medicine and Science, Los Angeles, CA USA; 2 University of Maryland Baltimore, Baltimore, MN USA; 3 Bethel University, Saint Paul, USA

**Keywords:** Academic Leadership, Physician Assistant, Medical Education, Doctoral degree, Gender, Minority, Program Director, Academic director, And Clinical Director

## Abstract

**Background:**

There is a critical need for a diverse pool of academic leaders to increase the number and diversity of the medical workforce. Physician Assistant/Associate (PA) is a growing medical profession. Although the master’s degree is the terminal degree for PAs, a growing number of PAs obtain a variety of doctoral degrees. However, there is no standardized training for academic PA leaders. The purpose of this study was to identify factors associated with PA academic leadership. Specifically, this study explored the following factors: doctoral degree credentials, gender and underrepresented minority status.

**Methods:**

Using the 2019 Physician Assistant Education Association Faculty and Directors survey, we assessed the relationship between academic leadership groups [Program Director (PD), Academic Director (AD), and Clinical Director (CD)] doctoral degree, gender, and underrepresented minority in medicine (URIM) status. Multivariable logistic regression models were used to determine the predictors of being in a leadership role. Results with p < 0.05 were considered statistically significant.

**Results:**

Of the 956 participants, 71% were female, 4% Hispanic, 86% White, 4% Black, 2% Asian, and 1% Native Hawaiian/Pacific Islander/American Indian/Alaska Native. Overall, 9% were URIM. Mean age was 45.6 (SD = 10.2) years. Average time in PA education was 2.9 years (SD = 1.4). Approximately 50% (n = 472) had a leadership role (PD-24%, AD-10%, CD-16%). Of all leaders, 68% were female, 9% were URIM, and 19% had a doctoral degree. Having a doctoral degree increased the odds of being a PD [AOR 2.38, CI [1.57–3.59], p = < 0.0001, AD and CD = non-significant]. More time in PA education increased the odds of being a PD [AOR 1.10, CI [1.07–1.12, p = < 0.0001] and AD [AOR 1.06, CI [1.03–1.09], p = < 0.0001], but not a CD. Gender and URIM status were not significantly associated with leadership roles. URIMs had doctorate degrees at higher rates than non-URIMs.

**Conclusion:**

PA academic leaders differ by doctoral degree attainment but not by gender and URIM status. URIM faculty are grossly underrepresented in the PA professorate, but disproportionately have doctoral degrees. Academic training opportunities for all PA academic leaders and strategies to increase URIM faculty are needed.

## Background

The foundation of any transformative healthcare workforce is largely shaped by those at the saddle of its professional training. Academic leadership is critically important to the physician assistant/associate (PA) profession which has undergone phenomenal growth and is expected to grow by an additional 31% by 2030[1]. Along with this growth, there comes leadership challenges such as appointing leaders with appropriate degree credentials and securing a diverse pool of PA academic leaders to administer these programs. Currently the factors associated with PA academic leadership are largely unknown. Specifically, there are no reports on how degree credentials, gender, and minority status influence leadership appointments in PA education.

In general, academic leadership is correlated with academic credentials [2, 3]. Unlike similar healthcare professions, the PA profession has not adopted doctoral level academic training. The master’s degree is the terminal degree designed to be optimally adequate training for clinical practice. There is currently no standardized pathway designed for PA professoriate and/or academic leadership, although essential competencies have been documented[4]. The profession nevertheless encourages advanced education, including doctoral degrees in any field of study[5]. As such, PA educators interested in doctoral degrees select from a wide variety of degree options including PhD, EdD and DHS, among others. A 2019 report indicated that overall, about 23.5% of all PA faculty and 45.5% of all PDs held a doctoral degree[6]. However, the prevalence of doctoral degree training among ADs and CDs remains unknown.

Along with academic credentials, gender and minority status are strong determinants in academic leadership [7–10], but whether this is true in the PA profession remains to be studied. It is well accepted that a diverse professoriate is associated with a diverse student body and ultimately, a diverse body of practitioners[11]. However, adequate representation of gender and racial minority faculty in academic leadership remains a challenge for most graduate-level professional education[12]. In 2019, 68% of all PA faculty (excluding medical directors -MDs & DOs) were female[6]. However, the distribution of gender within the academic leadership team remains unknown. In the same report, less than 10% of all PA faculty (excluding medical directors -MDs & DOs) were URIM[6]. URIM status is defined by the Association of American Medical Colleges as “those racial and ethnic populations that are underrepresented in the medical profession relative to their numbers in the general population”[13]. In this PAEA report, URIM status included African American, Native Hawaiian/Pacific Islander (NHPI), American Indian/Alaska Native (AIAN), and Hispanic ethnicity of any race.

The purpose of this study was to identify factors associated with PA academic leadership. Specifically, this study explored the following factors: doctoral degree credentials, gender and underrepresented minority status.

## Methods

Research Design and Participants: This study was a cross-sectional analysis of data obtained from the Physician Assistant Education Association (PAEA) 2019 Faculty and Directors survey. PAEA sent the survey to program directors at all 243 accredited PA programs in the US in July 2019. Program directors were instructed to distribute it to their core/principal faculty, and to provide a headcount for response rate calculation. Reminders were sent periodically to those who had not completed the survey until it closed in December 2019. The survey response rate was 60.5% with representation from 97.9% of all programs. PAEA performed data validation prior to giving the de-identified aggregate data to the researchers. For this study, only participants who reported ever having been certified as PAs were included. Faculty with less than 50% full time effort and adjunct faculty were excluded, since these individuals rarely hold leadership positions in PA programs and including them would bias the analyses. The Institution Review Board at Charles R. Drew University of Medicine and Science approved the study.

Dependent variable: PA academic training is structured into two core areas: didactic academic instruction and apprenticeship-type clinical training. Logistically, at least one faculty is dedicated to the administrative leadership of each of these areas, typically titled Academic Director (AD) or coordinator, and Clinical Director (CD) or coordinator. The overall program-wide administrative leadership is performed by a program director (PD). For most programs, this triad forms the core of the program’s administrative leadership. Therefore the dependent variable was self-reported leadership defined as a 4-category variable: PD (includes associate or assistant (PD), AD (or coordinator), CD (or coordinator), or no leadership (NL) if none of the above applied. We also investigated leadership as a 2-category (leadership yes/no) variable by combining PD, AD, and CD categories.

Main independent variables: Having a doctoral degree was defined as having any type of doctoral degree and was coded as yes/no. Gender was coded as either male or female and excluded other categories (n = 21). We examined the variable URIM, which characterizes the diversity of the medical workforce. URIM status was coded as yes/no.

Other independent variables: We looked at several additional independent variables that characterized the respondents and their roles as academic faculty. We report age (years), years in PA education, years in current position, and decade of first certification by the National Commission on Certification of Physician Assistants (NCCPA). Tenure status was categorized as having tenure, being in a tenure-track position but not having tenure, or not being in a tenure-track position. The survey included a question about whether participants ever published during their PA career, which was coded as yes/no. Participants were asked about receiving research funding from a variety of sources in the last 3 years. We constructed a single variable indicating whether the participant replied “yes” to any of the funding questions.

Data analysis: We characterized the sample using descriptive and bivariate analyses and tested for significance with chi-squared tests or ANOVA. To determine which predictors were associated with leadership in PA education, we ran multiple logistic regression models using both the 4-category and 2-category leadership outcome variables. Predictors in these models included our main independent variables, doctoral degree, gender, and URIM status, and we also controlled for number of years in PA education. We considered the inclusion of several time-dependent variables that may have had an independent association with being in a leadership position: age, number of years in PA education, number of years in current position, and decade since first certified. However, because these four variables were highly correlated, we opted to include just one in multivariable analyses to avoid destabilizing the parameter estimates. We selected number of years in PA education for theoretical reasons and because preliminary bivariate analyses indicated that it was strongly associated with leadership. We report odds ratios for unadjusted univariate and adjusted multivariable regressions. All analyses were performed using SAS 9.4. A p-value < 0.05 was used to determine statistical significance.

## Results

Participant Characteristics: Table 1 shows the characteristics of all participants. About 50% of all faculty were serving in a leadership role. Program directors made up about half of all leaders, and about 10% and 16% were ADs and CDs respectively. The mean age was 45 years, and, on average, participants had been in PA education for approximately 3 years and been at their current program slightly less (2.5 years). The largest proportion (43%) had received their NCCPA certification in the 2000s. The sample was mostly female (71%) and identified as white (86%). About 9% of participants were identified as having URIM status.

**Table 1:** Characteristics of Participants by Leadership Role

Characteristic	Total	No Leadership (NL)	Academic Director (AD)	Clinical Director (CD)	Program Director (PD)	P value
		**# (%)**	**# (%)**	**# (%)**	**# (%)**	
	**956**	**472 (49.4)**	**95 (9.9)**	**156 (16.3)**	**233 (24.4)**	
**Has a doctorate**						< 0.0001
No	771 (80.8)	407 (52.8)	76 ( 9.9)	142 (18.4)	146 (18.9)	
Yes	184 (19.3)	65 (35.3)	19 (10.3)	13 ( 7.1)	87 (47.3)	
**Decade of first NCCPA certification**						< 0.0001
1970s/80s	99 (10.7)	39 (39.4)	13 (13.1)	11 (11.1)	36 (36.4)	
1990s	222 (24.1)	83 (37.4)	23 (10.4)	30 (13.5)	86 (38.7)	
2000s	397 (43.1)	203 (51.1)	39 ( 9.8)	71 (17.9)	84 (21.2)	
2010s	204 (22.1)	131 (64.2)	17 ( 8.3)	37 (18.1)	19 ( 9.3)	
**Gender**						0.0279
Male	270 (28.8)	128 (47.4)	23 ( 8.5)	36 (13.3)	83 (30.7)	
Female	667 (71.2)	336 (50.4)	69 (10.3)	116 (17.4)	146 (21.9)	
**Race/Ethnicity**						
White	817 (85.5)	405 (49.6)	84 (10.3)	131 (16.0)	197 (24.3)	
Asian	19 (2.0)	10 (52.6)	1 ( 5.3)	4 (21.1)	4 (24.1)	
African American	38 (4.0)	17 (44.7)	6 (15.8)	6 (15.8)	9 (23.7)	
Hispanic	36 (3.8)	17 (47.2)	3 (8.3)	7 (19.4)	9 (25.0)	
NHPI/AIAN	8 (0.8)	4 (50.0)	0 (0.0)	1 (12.5)	3 (1.3)	
Other or no answer	38 (4.0)	19 (50.0)	1 (2.6)	7 ( 18.4)	11 (29.0)	
**Under-represented status in medicine**						0.9523
Non-UR in medicine	831 (91.0)	413 (49.7)	84 (10.1)	134 (16.1)	200 (24.1)	
UR in medicine	82 (9.0)	38 (46.3)	9 (11.0)	14 (17.1)	21 (25.6)	
**Tenure status**						< 0.0001
Not tenure track	762 (79.7)	384 (50.4)	74 ( 9.7)	130 (17.1)	174 (22.8)	
Tenure track, not tenured	137 (14.3)	67 (48.9)	19 (13.9)	24 (17.5)	27 (19.7)	
Tenured	57 (6.0)	21 (36.8)	2 ( 3.5)	2 ( 3.5)	32 (56.1)	
**Ever published**						< 0.0001
No	460 (49.4)	266 (57.8)	42 ( 9.1)	93 (20.2)	59 (12.8)	
Yes	472 (50.6)	195 (41.3)	51 (10.8)	56 (11.9)	170 (36.0)	
**Received research funding in last 3 years**						0.0357
No	819 (85.7)	412 (50.3)	87 (10.6)	132 (16.1)	188 (23.0)	
Yes	137 (14.3)	60 (43.8)	8 ( 5.8)	24 (17.5)	45 (32.8)	
	**Mean ± SD**	**Mean ± SD**	**Mean ± SD**	**Mean ± SD**	**Mean ± SD**	**p value**
**Age**	45.6 ± 10.2	46.3 ± 10.0	43.8 ± 9.8	44.0 ± 10.3	50.1 ± 9.5	< 0.0001
**Years in PA Education**	2.9 ± 1.4	3.2 ± 1.3	2.5 ± 1.3	2.6 ± 1.3	3.9 ± 1.1	< 0.0001
**Years at Current Program**	2.5 ± 1.3	2.6 ± 1.2	2.2 ± 1.3	2.3 ± 1.2	3.1 ± 1.2	< 0.0001

Leadership and Doctoral Degree Credentials: Overall, most participants (81%) did not have a doctoral degree (Table 1). Of those with doctoral degrees, 47% were PDs and 35% were not in a leadership position. 26% of males had doctoral degrees compared to 17% of females. Only 8% of CDs and 20% of ADs had doctoral degrees. In univariate analysis, there was a significant association between having a leadership role (all leadership roles combined) and having a doctoral degree [OR [95% CI] = 2.05 [1.47–2.86], p < 0.0001], which was reduced but remained significant after adjusting for years in PA education, gender and URIM status, [AOR [95% CI] = 1.46 [1.01–2.11], p = 0.0421] (Table 2).

**Table 2:** Unadjusted and adjusted odds ratios for predictors of having any leadership role

	Any leadership role (N = 904)			
	**OR [95% CI]**	**pvalue**	**AOR [95% CI]**	**pvalue**
**Doctorate: yes vs. no**	**2.05 [1.47–2.86]**	**< 0.0001**	**1.46 [1.01–2.11]**	**0.0421**
**Yrs in PA education**	**1.07 [1.05–1.09]**	**< 0.0001**	**1.07 [1.04–1.09]**	**< 0.0001**
**Gender: female vs. male**	0.89 [0.67–1.18]	0.4115	1.08 [0.80–1.47]	0.6158
**UR in Med vs. Non-UR in Med**	1.14 [0.73–1.80]	0.5631	1.05 [0.65–1.70]	0.8492

To further understand the relationship between being in a leadership role and having a doctorate degree, we compared this relationship within the three leadership types (Table 3). In univariate analyses, having a doctoral degree significantly increased the odds of having a PD role [OR [95% CI] = 3.73 [2.57–5.42], p < 0.0001]. However, having a doctoral degree did not change the odds of being in an AD or CD role. After adjusting for length of time employed in PA education, gender and URIM status, the significant relationship with having a doctorate degree remained for PDs [AOR [95% CI] = 2.38 [1.57–3.59], p < 0.0001]. Although there was not a statistically significant relationship between having a doctoral degree and having a CD role, there was an inverse trend in the multivariable model i.e., having a doctoral degree decreased the odds of being a CD [AOR [95% CI] = 0.56 [0.29–1.09], p = 0.0874].

**Table 3:** Unadjusted and adjusted odds ratios for predictors of having each type of leadership role

	OR [95% CI]	pvalue	AOR [95% CI]	pvalue
**Academic Director/Coordinator (N = 91)**				
**Doctorate: yes vs. no**	1.57 [0.89–2.76]	0.1224	1.23 [0.67–2.24]	0.5037
**Yrs in PA education**	**1.06 [1.03–1.10]**	**< 0.0001**	**1.06 [1.03–1.09]**	**0.0001**
**Gender: female vs. male**	1.14 [0.68–1.91]	0.6116	1.24 [0.73–2.09]	0.4247
**URIM vs. Non-URIM**	1.16 [0.54–2.51]	0.6968	1.06 [0.47–2.38]	0.8848
**Clinical Director/Coordinator (N = 146)**				
**Doctorate: yes vs. no**	0.57 [0.31–1.07]	0.0821	0.56 [0.29–1.09]	0.0874
**Yrs in PA education**	1.00 [0.97–1.03]	0.9633	1.01 [0.98–1.04]	0.5962
**Gender: female vs. male**	1.23 [0.80–1.88]	0.3468	1.32 [0.84–2.06]	0.2294
**URIM vs. Non-URIM**	1.14 [0.60–2.16]	0.6994	1.04 [0.53–2.06]	0.9111
**Program Director (PD) (N = 219)**				
**Doctorate: yes vs. no**	**3.73 [2.57–5.42]**	**< 0.0001**	**2.38 [1.57–3.59]**	**< 0.0001**
**Yrs in PA education**	**1.11 [1.09–1.14]**	**< 0.0001**	**1.10 [1.07–1.12]**	**< 0.0001**
**Gender: female vs. male**	**0.67 [0.48–0.94]**	**0.0205**	0.88 [0.61–1.29]	0.5233
**URIM vs. Non-URIM**	1.14 [0.65–2.00]	0.6444	1.04 [0.56–1.92]	0.9070

Leadership and Gender: In our sample, males and females held AD and CD leadership positions at similar rates, but males had higher rates of PD roles (p = 0.0279; Table 1), and this was further confirmed in multivariate analysis. Before adjustment, females had significantly lower odds of holding a PD position [OR [95% CI] = 0.67 [0.48–0.94], p = 0.0205], but this relationship was no longer significant after adjustment for doctoral degree, years in education, and URIM status (p = 0.5233; Table 3).

**Fig. 1:**
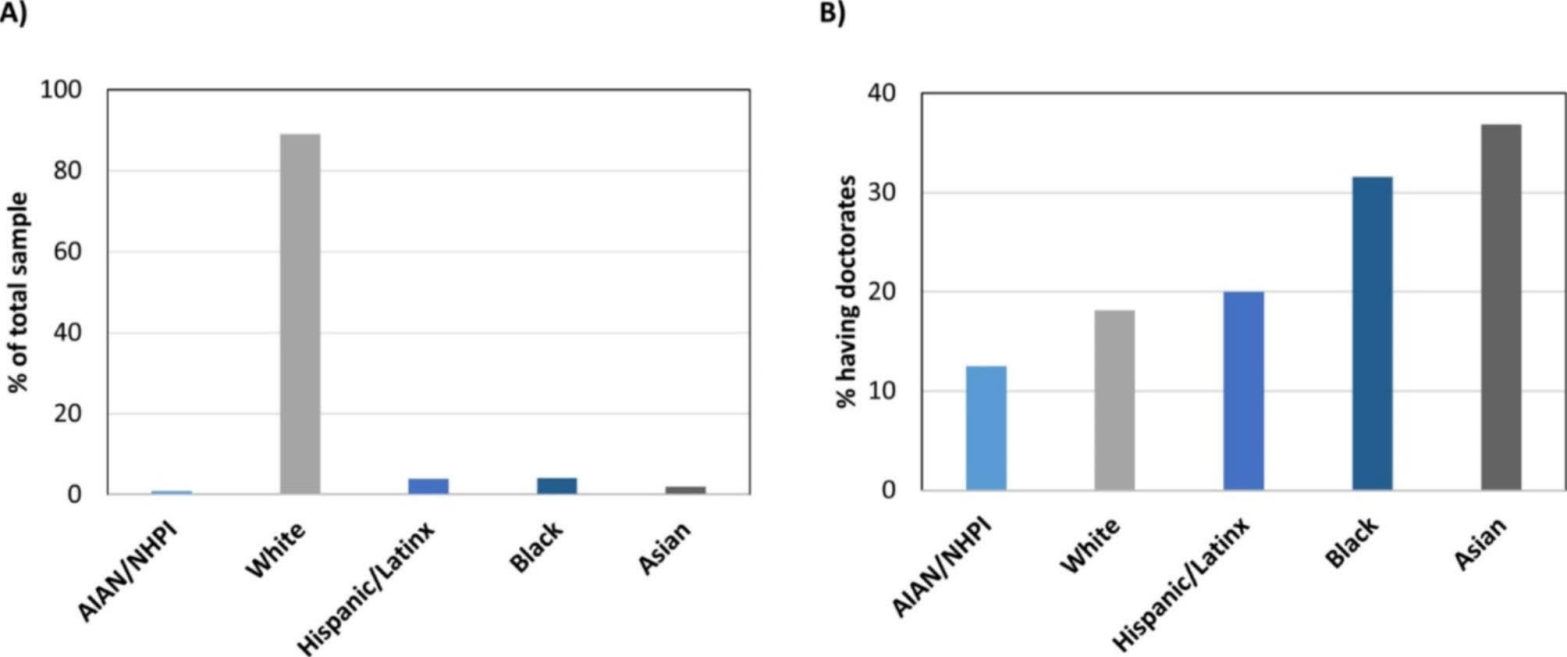
Representation of racial/ethnic group and doctoral degree holding among PA faculty. (A) The percent of the total sample identifying as each racial/ethnic group. (B) Percent of each racial/ethnic group having a doctoral degree

Leadership and URIM: There were no statistical differences among URIM and non-URIM in holding leadership positions (Table 1). However, further stratification of the URIM categories showed noteworthy patterns. Despite being a small proportion of the overall sample (Fig. 1 A), those identifying as URIM were proportionally represented among those with leadership positions. Additionally, the URIM participants identifying as Black or Hispanic had higher proportions of doctoral degrees than those identifying as white (Fig. 1B).

Other relevant findings: As expected, having spent more years in PA education increased the odds of being in a leadership role (Table 2). For every one year in PA education, the adjusted odds of being in any leadership role increased by 7% [95% CI = 4–9%]. This association held for both ADs and PDs but was strongest for PDs (Table 3). For every year in PA education, the adjusted odds of being an AD increased by 6% [95% CI = 3–9%], and the adjusted odds of being a PD increased by 10% [95% CI = 7–12%]. There was no significant relationship between years in PA education and having a CD role. Similarly, in bivariate analysis (Table 1), longevity in the PA profession (related or unrelated to academia), based on when the participant first became NCCPA certified, was significantly associated with leadership role, with PDs and ADs tending to have had longer careers. However, among the leaders, the majority of those most recently certified held the CD position. Those in the PD group were in PA education and at the current program longer than ADs and CDs.

Overall, most participants (80%) were not tenured or in a tenure track. About 50% had had a publication, but only 14% received research funding in the previous 3 years. Although the numbers were low, those who were tenured, had published and had received research funding were mostly PDs and NLs.

## Discussion

This study examined the factors associated with PA academic leadership positions (PD, AD, and CD). Specifically we investigated doctoral degree credentials, gender, and underrepresented minority status. Program directors were more likely to have an advanced degree and tended to have been in PA education longer than other leadership cadres and faculty. Gender and URIM status were equally represented in all leadership roles within the PA professoriate. To our knowledge, this is the first study reporting on the role of doctoral degree credentials, gender, and minority status within academic leadership among PA-trained faculty.

Leadership and Doctoral Degree Credentials: Our study highlights the limited supply (19%) of doctoral-trained PA faculty overall, and the disproportionate distribution of advanced academic credentials among faculty. Having a doctoral degree increased the odds of being in a program director position two-fold. The direction of this relationship is unclear, i.e., does obtaining a doctoral degree create opportunities for PD roles, or do those already in the PD role prospectively obtain the doctoral degree? A combination of both pathways is likely. A recent study reported an employer preference for doctoral-trained faculty, particularly for program director positions[3, 14]. This trend may compel those interested in advancing their professoriate career to obtain a doctoral degree to be competitive on the job market. The recent growth of doctoral programs earmarked for PAs[15] has provided these opportunities. For those already holding leadership positions, there are multiple factors that drive them to obtain doctoral degrees. In most academic institutions, tenure, promotion, and seniority are largely dependent on academic credentials. Indeed, PDs were more likely to have published, received grants and have tenure compared to ADs and CDs. Additionally, interaction with doctoral-trained academic leaders from other similar programs may influence the desire to pursue doctoral education. Regardless of the direction of this relationship, it remains to be established whether doctoral degree training improves PA program director leadership competencies or enhances program wide outcomes[16].

The disparity in doctoral degree credentials between PDs and other leaders may be explained by the accompanying finding of ADs and CDs having the least longevity in PA education (2.5 years) and therefore may be still in a status of rapid adjustment and equilibrium. Additionally, a doctoral degree requires an investment of time, financial and personal sacrifices without a guarantee for a positive return on investment[3, 14] therefore limiting the motivation/incentive for this cadre of leaders to further their education to the doctoral level. Moreover, the skillsets for AD and CD roles are largely focused on PA curriculum and pedagogical acumen, student performance and success, and internal and external relationship building. Whist similar across programs, these skillsets are customized and mastered within each academic program. Moreover, most doctoral degrees obtained by PAs do not provide specialized training[17] in PA education and therefore may not be regarded as valuable to their current roles. Instead, the PAEA offers focused and relevant trainings for ADs and CDs. Therefore, those in these positions may not be motivated to pursue formal academic doctoral degrees. Among NLs, preclusion from leadership burden, and therefore more time to pursue further education may explain the proportion of NLs with doctoral degrees. Perhaps for the same reason, non-leaders reported more academic scholarship than leaders.

Leadership and Gender: Previous studies have reported significant differences between the numbers of females and males in senior leadership positions in academic medicine within the US[7, 8]. After controlling for several variable, there were no gender differences in the odds of having any leadership role in this study. Notably however, more male faculty have doctoral degrees compared to female faculty.

Leadership and URIM: Our findings highlight the scarcity of faculty from backgrounds underrepresented in medicine. In our sample of over 900 PA faculty, only 38 (4.0%) were African American, only 36 (3.8%) were of Hispanic origin, and only 8 (0.8%) were AIAN/NHPI. This trend is consistent with the national PA census and matriculation statistics. In 2020, among certified PAs, 3.3% identified as African American, 6.7% as Hispanic, and 0.7% AIAN/NHPI[18]. Because applicant matriculation provides the pipeline for future PAs in practice, and in faculty roles, disparities in matriculation directly impact disparity in faculty diversity. This is evident in the composition of matriculating students in 2017-18 (graduation ~ 2020). Only 3.8% of all matriculants were African American and 0.5% AIAN/NHPI (compared to 80% White), and 8.8% were Hispanic [19]. While there are many factors associated with these disparities, there is a critical need for strategies to increase minority student enrollment [20, 21].

Because of these disparities, our URIM sample was small (n = 82). However, despite the URIM faculty being only a small fraction of the total sample, they were overrepresented among those with doctoral degrees. Reasons for this finding are unclear. It is plausible that the few URIM faculty are more likely to have predictors of leadership competencies, a self-selection and self-drive for leadership affinity [9]. This observation could be driven by the long-lasting racial imbalances that have existed in the US, leading to URIM faculty to feel the need to overachieve to succeed in academic leadership. This “working twice as hard” phenomenon has been reported in a higher education leadership qualitative study [10]. In this study that examined women of color in faculty governance, participants reported having to go the extra mile to be seen as credible and capable. For example, they felt the need to take on more roles beyond their non-URIM counterparts. For similar reasons, URIM faculty may feel the pressure to obtain doctoral degrees in order to be considered for leadership positions, while non-URIM faculty may not have a similar perception. Future qualitative studies will shed more light on this observation.

Strengths: Our study has several strengths. First, our analyses included only faculty who identified as PAs and principal faculty at > 50%FTE. Second, the dataset represented participants from 97.9% of PA all PA programs in the country. Third, our analyses and study design looked at the differences within the PA leadership team by roles.

Limitations: Because of the cross-sectional design of this study, the direction of the relationships we found cannot be inferred and data on other non-academic leadership training was not available to be considered. For faculty with doctoral credentials, the survey did not inquire if the doctoral degree was obtained before or after becoming a PA. Additionally, self-reported survey data is subject to individual bias and misreporting and some respondents did not answer all survey questions. The proliferation of doctoral degree offerings in the PA education landscape may limit the generalizability of these findings. Our sample size did not allow detailed analysis regarding the types of doctoral degrees held by faculty, but this is an avenue for further research. Finally, these data were collected in Spring 2019, just before the COVID-19 pandemic; resultant workforce dynamics may change how leadership is associated with doctoral degree credentials, gender, and minority status post pandemic.

## Conclusion

In summary, the likelihood of being a PA academic leader differs by terminal degree, but not by gender and URIM status. This study identifies three main differences among the PA professorate. First, ADs and CDs, although members of the PA academic leadership team, do not have advanced academic credentials as do PDs. Second, of all leaders, PDs have more longevity in academia. And third, URIM faculty are grossly underrepresented in the PA professoriate, but the few URIM faculty have received advanced academic credentials. Institutions could adopt a “grow your own” strategy by offering protected time, tuition re-imbursement and other incentives for doctoral training. Investing in all faculty to obtain advanced degrees can offer high return on investment for PA programs as it may improve leadership pipeline, improve teaching, recruitment, grants and scholarship, as well the overall organization success. At the institutional level, academic parity with other leaders may open doors for expanded roles, scholarship, and *interprofessional* collaboration. Increasing URIM faculty needs to start at the PA applicant level by adopting strategies to increase URIM PA applicants and increase matriculation rates. Strategies to train current URIM faculty to prevent departure and train practicing PAs for academic roles are needed.

## Data Availability

The datasets used in the current study are available from the Physician Assistant Education Association through a formal request.

## Declarations

**Ethic approval and consent to participate**.

Institution Review Board at Charles R. Drew University of Medicine and Science approved this secondary analysis of de-identified data.

## List of abbreviations

PA: Physician assistant/associate
PAEA: Physician Assistant Education Association
PD: Program director
AD: Academic director
CD: Clinical director
URIM: Underrepresented minority in medicine

## References

[1] *Occupational Outlook Handbook: Physician Assistants*. 2021 03/20/2022]; Available from: https://www.bls.gov/ooh/healthcare/physician-assistants.htm#:~:text=in%20May%202020.-,Job%20Outlook,on%20average%2 C%20over%20the%20decade.

[2] Mertens A, Röbken H (2013). Does a doctoral degree pay off? An empirical analysis of rates of return of German doctorate holders. High Educ.

[3] Kayingo G (2022). Assessing demand for doctoral-prepared PA faculty: a five-year longitudinal study. BMC Med Educ.

[4] Zaweski J (2019). Physician Assistant Educator Competencies. J Physician Assistant Educ.

[5] *Physician Assistant Clinical Doctorate Summit: Final Report and Summary*. The Journal of Physician Assistant Education, 2009. 20(2): p. 22–22.

[6] *Physician Assistant Education Association, By the Numbers: Faculty Report 4: Data from the 2019 Faculty & Directors Survey. Washington, DC: PAEA; 2020. 10.17538/FR4.2020* 2020.

[7] Schor NF (2018). The Decanal Divide: Women in Decanal Roles at U.S. Medical Schools. Acad Med.

[8] Sklar DP (2018). Leadership in Academic Medicine: Purpose, People, and Programs. Acad Med.

[9] Isik U (2021). As an ethnic minority, you just have to work twice as hard.“ Experiences and motivation of ethnic minority students in medical education. Perspect Med Educ.

[10] Reis NM (2010). Why are there so few of us? Counterstories from women of color in faculty governance roles. Educational Leadersh.

[11] Mitchell KM (2021). Strategies for retention of nursing students: A scoping review. Nurse Educ Pract.

[12] Association of American Medical Colleges. Diversity in medicine: facts and Fig. 2019. Association of American Medical Colleges Washington, DC; 2019.

[13] AAMC. *Underrepresented in Medicine Definition*. 03/22/2022]; Available from: https://www.aamc.org/data-reports.

[14] Kayingo G (2021). Demand and supply for doctoral-prepared PA faculty: A 5-year national longitudinal study. JAAPA.

[15] Kibe L. *Growing pains have no boundaries*. Journal of the American Academy of Physician Assistants, 2017. 30(11).

[16] Bushardt RL (2012). Physician assistant program characteristics and faculty credentials on physician assistant national certifying exam pass rates. J Physician Assist Educ.

[17] Kibe LW, Kayingo G. *Developing Doctoral Programs for PA Educators: Is It Time?* 2020 04/15/2020]; Available from: https://paeaonline.org/resources/public-resources/paea-news/developing-doctoral-programs-for-pa-educators-is-it-time.

[18] NCCPA. *Statistical Profile of Certified PAs Annual Report*. 2020 04/15/2022]; Available from: https://www.nccpa.net/wp-content/uploads/2021/07/Statistical-Profile-of-Certified-PAs-2020.pdf.

[19] PAEA. *The PA Pipeline to Practice Applicant and Matriculation Data from CASPA* 2020.

[20] Coplan B, Bautista TG, Dehn RW (2018). PA program characteristics and diversity in the profession. JAAPA.

[21] Parkhurst DC, Kayingo G, Fleming S (2017). Redesigning Physician Assistant Education to Promote Cognitive Diversity, Inclusion, and Health Care Equity. J Physician Assist Educ.

